# Multiple, non-allelic, intein-coding sequences in eukaryotic RNA polymerase genes

**DOI:** 10.1186/1741-7007-4-38

**Published:** 2006-10-27

**Authors:** Timothy JD Goodwin, Margaret I Butler, Russell TM Poulter

**Affiliations:** 1 Department of Biochemistry, University of Otago, P.O. Box 56, Dunedin, New Zealand

## Abstract

**Background:**

Inteins are self-splicing protein elements. They are translated as inserts within host proteins that excise themselves and ligate the flanking portions of the host protein (exteins) with a peptide bond. They are encoded as in-frame insertions within the genes for the host proteins. Inteins are found in all three domains of life and in viruses, but have a very sporadic distribution. Only a small number of intein coding sequences have been identified in eukaryotic nuclear genes, and all of these are from ascomycete or basidiomycete fungi.

**Results:**

We identified seven intein coding sequences within nuclear genes coding for the second largest subunits of RNA polymerase. These sequences were found in diverse eukaryotes: one is in the second largest subunit of RNA polymerase I (*RPA2*) from the ascomycete fungus *Phaeosphaeria nodorum*, one is in the RNA polymerase III (*RPC2*) of the slime mould *Dictyostelium discoideum *and four intein coding sequences are in RNA polymerase II genes (*RPB2*), one each from the green alga *Chlamydomonas reinhardtii*, the zygomycete fungus *Spiromyces aspiralis *and the chytrid fungi *Batrachochytrium dendrobatidis *and *Coelomomyces stegomyiae*. The remaining intein coding sequence is in a viral relic embedded within the genome of the oomycete *Phytophthora ramorum*. The *Chlamydomonas *and *Dictyostelium *inteins are the first nuclear-encoded inteins found outside of the fungi.

These new inteins represent a unique dataset: they are found in homologous proteins that form a paralogous group. Although these paralogues diverged early in eukaryotic evolution, their sequences can be aligned over most of their length. The inteins are inserted at multiple distinct sites, each of which corresponds to a highly conserved region of RNA polymerase. This dataset supports earlier work suggesting that inteins preferentially occur in highly conserved regions of their host proteins.

**Conclusion:**

The identification of these new inteins increases the known host range of intein sequences in eukaryotes, and provides fresh insights into their origins and evolution. We conclude that inteins are ancient eukaryote elements once found widely among microbial eukaryotes. They persist as rarities in the genomes of a sporadic array of microorganisms, occupying highly conserved sites in diverse proteins.

## Background

An intein (internal protein) is a protein sequence that is translated as an insertion within a host protein. The intein is then post-translationally excised, simultaneous with the ligation of the two flanking segments of the host protein [1-7]. The result of intein excision is two proteins derived from a single initial translation product: (i) the free intein sequence, and (ii) the mature form of the host protein, with the two halves (the N-terminal and C-terminal external proteins, or exteins) ligated by a peptide bond. The reactions in which the intein is excised from the precursor protein and the flanking exteins are joined are mediated primarily by the intein itself, although the first residue of the C-extein also has an important role. The term intein strictly refers to a protein molecule, but the gene segment encoding the intein is also often referred to as an intein.

In addition to containing sequences necessary for their excision and the splicing of their flanking exteins, many inteins have a homing endonuclease domain. Inteins carrying such domains are often referred to as full-length inteins. Some inteins lack a homing endonuclease domain, containing only those sequences necessary for their excision and extein splicing. These are known as mini-inteins. Most of the homing endonuclease domains found in full-length inteins belong to the LAGLIDADG family [5]. Homing endonucleases are believed to promote the spread of an intein through the gene pool of the host species via a recombination process (homing). In a diploid cell heterozygous for the intein, cleavage of the empty allele by the homing endonuclease will be followed by DNA repair performed by the host repair machinery, using the occupied allele as a template [8]. This will result in the cell becoming homozygous for the intein. In this way, the intein gene is duplicated and can spread throughout a population. Most inteins have no known function, and thus are considered to be selfish or parasitic elements [9]. However, inteins are efficiently removed from the host protein [10-14], so their effect on the host phenotype is minimal.

The homing pathway is dependent on the homing endonuclease recognition of the target site and on the allelic homology of the surrounding sequences. If an intein homing endonuclease were to cut an ectopic site, this would not precipitate homologous recombination (gene conversion) of the intein sequence because of the lack of flanking homology. For this reason, it is apparently very difficult for inteins to move to (or colonise) a new site, and such ectopic movement is likely to be a very rare event. This belief is supported by the finding that allelic inteins (i.e. inteins inserted at corresponding sites in homologous genes), even in distantly related species, are usually more closely related to each other than they are to non-allelic inteins, including those from the same species [5,9,15].

Inteins are rarities, and have a puzzling distribution among genes and species: the majority of species do not carry any known inteins, while some species have many; for example, the archaeon *Methanococcus jannaschii *has 19 distinct inteins. The species that carry inteins do not cluster together on evolutionary trees, but are phylogenetically dispersed, and closely related species do not necessarily have similar sets of inteins. Inteins have only been found in microorganisms. The vast majority of genes have no known inteins, but some genes contain multiple inteins. For instance, replication factor C of *M. jannaschii *contains three distinct inteins [16] and a ribonucleotide reductase of *Trichodesmium erythraeum *contains four [13]. Of the more than 80 distinct (non-allelic) inteins described, most (>75%) are found in genes involved in replication or transcription, such as DNA polymerases and helicases, or in related processes such as the metabolism of nucleotides (together these genes could be said to have information-processing functions).

The reasons behind the unusual distribution of inteins are currently unknown. One possible explanation for their phylogenetic distribution is that inteins were formerly much more widespread than they are now, but over time they have been randomly lost on many independent occasions in different lineages, resulting in their current sporadic appearances [9]. It is also possible that their distribution is partly a result of horizontal transfer (that is, movement between species that might be only distantly related). The predominance of inteins in information-processing genes may reflect the horizontal transfer of inteins via virus infection [17]. The genomes of phage and viruses consist predominantly of genes involved in information processing. It is possible that the pattern of multiple coincident insertions is also a reflection of the inteins occurring predominantly in the subset of genes that are common to cellular organisms and their infecting viruses. Three of the allelic intein groups have members that are genomic and viral. For example, RIR1-l allelic inteins are found in eubacteria, eubacterial phages and the eukaryote iridescent viruses, DnaB-b allelic inteins are present in eubacteria and their phages, while Pol-c allelic inteins are found in archaea and in eukaryote viruses (mimivirus and the *Heterosigma akashiwo *virus (HaV)).

In total, five distinct inteins have been found in eukaryotic nuclear genes. These appear in the *VMA1 *gene that encodes a subunit of a vacuolar membrane adenosine triphosphatase [10,18]; *PRP8*, encoding an essential component of the spliceosome [19]; *GLT1*, glutamate synthase [20]; *CHS2*, chitin synthase 2 [20]; and *ThrRS*, threonyl tRNA synthetase (submitted by S. Pietrokovski to InBase [6]). All of these nuclear-encoded inteins have been found exclusively in fungi. VMA inteins have been found in a variety of hemiascomycete yeasts, including *Saccharomyces cerevisiae*, *Kluyveromyces lactis *and *Candida tropicalis*. The PRP8 intein was first found in the basidiomycete fungus *Cryptococcus neoformans *[19]. Since then, PRP8 inteins have been found in some additional *Cryptococcus *species (*C. gattii *and *C. laurentii*) [21] and in a variety of ascomycete fungi, including *Aspergillus fumigatus*, *Histoplasma capsulatum *and *Botrytis cinerea *[14,22] and in three species of *Penicillium *[23]. GLT1 inteins have been identified in a small number of ascomycetes (*Debaryomyces hansenii*, *Pichia guilliermondii*, *Podospora anserina *and *Phaeosphaeria nodorum*). The CHS2 intein has been found in only one species, *P. anserina*, despite a large number of fungal CHS2 gene sequences being available in GenBank. Finally, the fifth eukaryotic nuclear full-length intein gene, ThrRS, was very recently identified in the ascomycete yeast *C. tropicalis *(Pietrokovski, InBase [6]). An allelic mini-intein is also found in the closely related yeast *Candida parapsilosis*. In addition to these nuclear intein genes, three intein genes have been found in chloroplast genomes: there are allelic inteins in the DnaB helicase genes of the chloroplasts of the cryptophyte alga *Guillardia theta *[24] and the red alga *Porphyra purpurea *[25], and a distinct intein in the ClpP protease gene of the chloroplasts of the green alga *Chlamydomonas eugametos *[26,27]. Furthermore, inteins have been identified in viruses of eukaryotes: allelic inteins have been found in the DNA polymerase B genes of *Acanthamoeba polyphaga *mimivirus [28] and HaV01 [17]. A distinct full-length intein appears in the *RIR1 *gene of *Chilo *iridescent virus [29,30], with two other insect iridoviruses (*Costelytra zealandica *iridescent virus and *Wiseana *iridescent virus) containing allelic mini-inteins (Pietrokovski, InBase [6]). We have detected an intein in a helicase of PBCV (*Paramecium bursaria Chlorella *virus; PBCV) NY2A that is not present in the homologous sites of other PBCV strains (authors' unpublished data and InBase [6]).

DNA-dependent RNA polymerases are complex proteins consisting of several polypeptides including two large and several smaller subunits [31]. Eukaryote nuclei generally encode three RNA polymerases: RNA polymerase I synthesizes a pre-rRNA, 45S, which matures into 28S, 18S and 5.8S rRNAs that will form the major RNA sections of the ribosome. RNA polymerase II synthesizes precursors of messenger RNAs and most small nuclear RNAs. RNA polymerase III synthesizes transfer RNAs, 5S ribosomal RNAs and other small RNAs found in the nucleus and cytoplasm. Some of the various subunits of the different RNA polymerases (including the two largest subunits) are encoded by genes that are homologous (paralogous) throughout cellular life. Some viruses also contain homologous genes encoding their own RNA polymerase.

Here we report the identification and characterisation of seven previously undetected intein-coding sequences from eukaryotic nuclear genomes. These were all identified in genes encoding the second largest subunits of RNA polymerase. They are inserted at six distinct (non-allelic) sites. Four were found in fungi (an ascomycete, a zygomycete and two chytrids), one was found in the slime mould *Dictyostelium discoideum*, and one in the green alga *Chlamydomonas reinhardtii*. The last was an intein identified in a viral remnant embedded in the nuclear genome of the oomycete *Phytophthora ramorum*. Partial sequences of inteins allelic to this latter intein were also identified in the RNA polymerase of a strain of the *Emiliania huxleyi *virus and in a sequence generated by the Sargasso Sea Metagenomics Project. Analysis of these intein sequences leads to insights into the origins and evolution of inteins in eukaryotes.

## Results

### Identification of new nuclear intein genes

To identify new eukaryotic intein genes, we used the sequences of a wide variety of previously identified inteins (of eukaryotic, prokaryotic and viral origins) to perform BLAST searches of the publicly available eukaryotic sequence databases (including GenBank, and genome sequencing centre databases containing data not yet released to GenBank; see Methods section). High-quality matches identified in the BLAST searches, putatively representing new inteins, were then examined in detail for features characteristic of inteins [15]. For example, most inteins begin with a Cys or a Ser residue, end with the dipeptide His-Asn, and are followed by a Cys, Ser or Thr residue as the first amino acid of the C-extein. Inteins contain a number of conserved motifs associated with splicing, and most inteins contain a homing LAGLIDADG endonuclease domain. Inteins also appear as specific inserts within other proteins, and often appear at highly conserved sites within highly conserved proteins. Another important indicator of the presence of an intein encoding sequence is that the sequence is present in some homologues of the host gene, but is absent in most. Table 1 summarises the novel inteins described in this report.

### An RNA polymerase I intein in *Phaeosphaeria nodorum*

**Figure 1:**
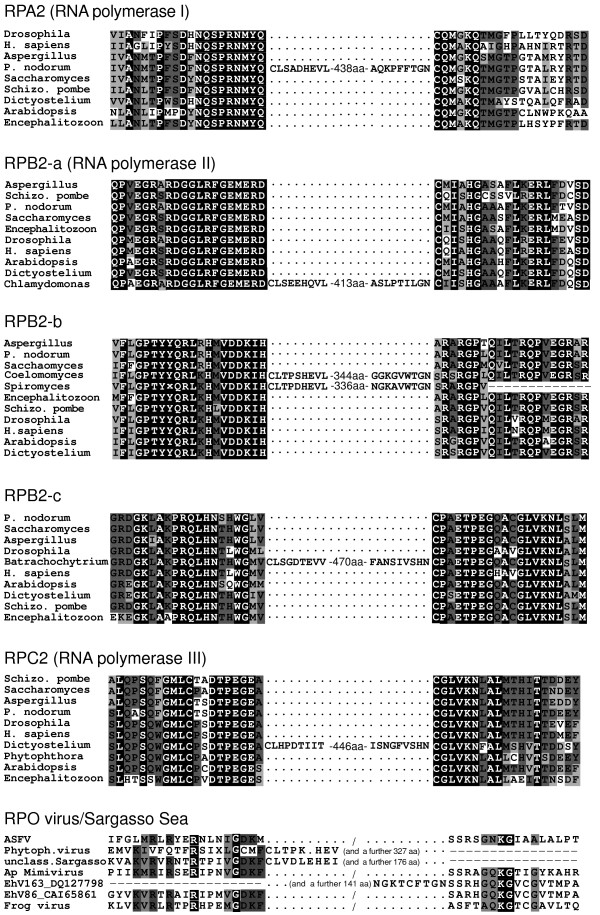
**Intein insertions into eukaryotic and viral RNA polymerases**. Alignments of intein/extein borders for the eight inteins in the six RNA polymerase intein insertion sites. RNA polymerase sequences are taken from accession data as described in Methods. The unclassified Sargasso Sea sequence is from GenBank accession AACY01369547, the *E. huxleyi *virus 163 sequence is from GenBank accession (DQ127798). The dashes represent missing data.

**Figure 2:**
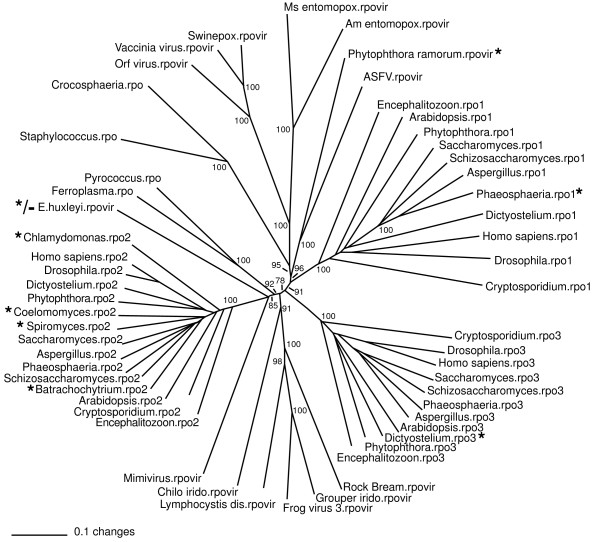
**Phylogenetic distance tree of RNA polymerases**. RNA polymerase sequences are taken from accession data as described in the Methods section. The unrooted tree was constructed by the neighbour-joining method using PAUP*4b10 [52] and the default settings. Numbers on the branches indicate the percentages of bootstrap support indicated by a heuristic search with 100 random addition replicates and the tree-bisection-reconnection branch-swapping algorithm. All bootstrap values > 50 have been reported except where they occur within the three well-supported RNA polymerase I (rpo1), RNA polymerase II (rpo2) and RNA polymerase III (rpo3) groups. RNA polymerases that contain an intein are indicated by asterisks (*); strains of the *E. huxleyi *virus are polymorphic for the presence of an intein in RNA polymerase (*/-). The alignment used is available as supplementary data (additional file 1).

*P*. (*Stagonospora*) *nodorum *is a filamentous ascomycete belonging to the class Dothideomycetes. It is a major pathogen of wheat crops. Strain SN15 has been sequenced by the whole genome shotgun method to >10-fold coverage at the Broad Institute [32]. The assembled sequence has been made publicly available in GenBank. Using the sequence of the *C. eugametos *chloroplast Ceu ClpP intein as a query in a TBLASTN search of the whole genome shotgun (WGS) sequence division of the GenBank database we detected a high quality match in *P. nodorum *sequence AAGI01000064 (E = 3 × 10^-6^; bases 49105–50471). This sequence can also be found on supercontig 1.4 (from base pair 1094221 to 1095587) at the Broad Institute website [32]. The matching region appears as an insert within a gene encoding the second largest subunit of RNA polymerase (Figure 1). Sequence comparisons (Figure 1) and phylogenetic analyses (Figure 2, additional file 1) indicate that the gene encodes a subunit of RNA polymerase I. The insert has numerous features indicating that it encodes an intein. It appears as an insertion within the RNA polymerase gene and consists of an uninterrupted open-reading frame (ORF) encoding 455 amino acids, in phase with the RNA polymerase ORF. The insert begins with a Cys residue and is followed by a Cys residue. The N- and C-terminal regions of the insert contain sequences corresponding to the conserved splicing domains of inteins (alignments of conserved intein domains can be viewed at the InBase website [6]). The central region contains the characteristic motifs of an LAGLIDADG homing endonuclease domain, although this appears to be degenerate and is unlikely to be still active. An unusual feature of this new intein is that it ends with the dipeptide Gly-Asn rather than the more common His-Asn. Gly-Asn termini have, however, been identified previously in inteins, for instance, in Ceu ClpP and in the ThrRS inteins of *C. tropicalis *and *C. parapsilosis*. This new intein has been named Pno RPA2, following intein naming conventions. It is the first intein to be identified in a gene encoding the second largest subunit of an RNA polymerase. The only previously identified RNA polymerase inteins (the archaeal inteins Mja rPol A' and Mja rPol A" from *M. janaschii *and Nph rPol A" from *Natronomonas pharaonis*) all appear in archaeal homologues of the gene encoding the largest subunit of eukaryotic RNA polymerase.

Note that the sequence AAGI01000064 has a frameshift in the region corresponding to the intein. Comparisons (not shown) with the *P. nodorum *sequences in the GenBank trace archives, however, suggest that this is a sequencing error resulting from the insertion of a G residue at position 50225 and a C at 50260. These were removed to generate the full sequence of the RNA polymerase gene with an uninterrupted ORF.

### An RNA polymerase II intein in *Chlamydomonas reinhardtii*

*C. reinhardtii *is a unicellular green alga. An intein in this species was first detected in several *C. reinhardtii *expressed sequence tag (EST) sequences using a TBLASTN search of the GenBank EST databases with the Ctr ThrRS intein sequence as a query. A full-length sequence of the intein was then retrieved from version 2 of the *C. reinhardtii *genome sequence assembly, available from the Joint Genome Institute [33]. The intein lies on scaffold 5, contig 26 (bases 289701–290993 on the minus strand). The intein, Cre RPB2, appears as an uninterrupted ORF encoding 431 amino acids inserted within the coding region of the *C. reinhardtii *gene for the second largest subunit of RNA polymerase II (Figures 1, 2). Like many other inteins, it begins with a Cys residue, is followed by a Cys residue in the C-extein, and contains the conserved splicing domains and an LAGLIDADG homing endonuclease domain (see InBase for alignments [6]). Like the Pno RPA intein, Cre RPB2 ends with a Gly-Asn dipeptide rather than the more common His-Asn. This is the first intein encoded in a nuclear genome to be found outside of the fungi.

### Further RNA polymerase II inteins

Three further inteins have been found in genes encoding the second-largest subunits of RNA polymerase II. The sequences of these genes were generated as part of the Assembling the Fungal Tree of Life (AFTOL) project [34], which is using, among other things, RNA polymerase II sequences to assist in determining the relationships among a wide variety of fungi. Inteins appear in *RPB2 *sequences from *Spiromyces aspiralis *(DQ302790), a zygomycete fungus, and *Coelomomyces stegomyiae *(DQ302766) and *Batrachochytrium dendrobatidis *(DQ302769), both members of the Chytridiomycota. These inteins again have the conserved splicing and endonuclease domains characteristic of inteins (see InBase [6]). They also appear as inserts within the RNA polymerase sequences. The *C. stegomyiae *intein, Cst RPB2, and the *S. aspiralis *intein, Sas RPB2, are inserted at homologous sites and are therefore allelic inteins. The *B. dendrobatidis *intein is inserted at a different site. Both of these sites are distinct from the insertion site of the *C. reinhardtii *RNA polymerase II intein, Cre RPB2. To distinguish the three intein insertion sites in RNA polymerase II genes they have been denoted "a", "b" and "c", according to the order in which they were identified: Cre RPB2 is in the "a" site, Cst RPB2 and Sas RPB2 are in the "b" site, and Bde RPB2 is in the "c" site. The allelic RPB2-b inteins are present in two very distantly related species, a zygomycete and a chytrid.

### An RNA polymerase III intein in *Dictyostelium discoideum*

*D. discoideum *is a slime mould classified within the Mycetozoa. The whole genome sequence has been determined and described [35]. An intein in *D. discoideum *was detected as an insertion of 464 amino acid residues within the second largest subunit of RNA polymerase III (Figures 1, 2; GenBank protein ID no. EAL63250). The intein, Ddi RPC2, appears as a specific insert within the RNA polymerase subunit relative to homologues from other species, and it is inserted at a different site from the *P. nodorum *RNA polymerase I intein and from any of the RNA polymerase II inteins. The *Dictyostelium *intein begins with a Cys residue, ends with a standard His-Asn dipeptide and is followed by a Cys residue. The N- and C-terminal parts contain the conserved splicing domains characteristic of inteins [6], while the central region contains a possibly degenerate LAGLIDADG homing endonuclease. Ddi RPC2 contains several low-complexity regions or short runs of the same amino acid. For instance, it contains a region of 13 amino acid residues, of which 11 are Asn residues. It also contains a region with seven consecutive Asn residues and two regions with seven consecutive Gln residues. Such low-complexity regions appear to be common features in *D. discoideum *proteins [35]. In the *D. discoideum *RNA polymerase III protein, they are restricted to the segment corresponding to the endonuclease domain of the intein, i.e. they are not found in the intein splicing domains or in the RNA polymerase sequence.

### An RNA polymerase intein in a viral remnant within the *Phytophthora ramorum *genome

*P. ramorum *is a member of the oomycetes, belonging to the kingdom Stramenopiles, which also includes diatoms, golden-brown algae and brown algae [36]. The genome sequence has been determined by the Joint Genome Institute [33,37]. Using the Ceu ClpP intein as a query in a TBLASTN search, we detected a high quality match (E = 3.0 × 10^-15^) in the *P. ramorum *genome (scaffold 19, bases 14734–15744 on the minus strand). This sequence has numerous features suggesting that it is an intein. For instance, it begins with a Cys residue and contains sequences similar to the splicing domains of other inteins (not shown). These are separated by a region containing an LAGLIDADG homing endonuclease domain similar to that of previously identified inteins. Immediately upstream of this putative intein is a long ORF encoding a putative protein homologous to the second largest subunit of RNA polymerase. The site at which the putative intein interrupts the RNA polymerase ORF is highly conserved, although it is distinct from the insertion sites of the previously identified RNA polymerase inteins (Figure 1).

In addition to having these similarities to other inteins, this putative intein has unusual features. Firstly, instead of being an uninterrupted ORF, the region encoding the intein-like sequence contains two frameshift mutations, which result in the appearance of stop codons within the coding reading frame. Secondly, although it contains most of the conserved motifs associated with intein splicing, it lacks the conserved residues (usually a His-Asn dipeptide) corresponding to the extreme C-terminal ends of inteins; instead, the corresponding sequence consists of a stop codon and an Arg codon (see additional file 2). These features suggest that the sequence no longer represents a functional intein (comparisons with sequences in the trace archives suggest that most of these are genuine mutations, although one of the frameshifts within the intein is likely to be a sequencing error; data not shown). Likewise, the RNA polymerase gene, in which the putative intein gene is inserted, has some unusual features. Firstly, it also appears to be non-functional; about 780 bp upstream of the intein insertion site, the RNA polymerase coding sequence contains a frameshift mutation and there is a nonsense mutation six codons upstream of the putative intein. Secondly, the section of the RNA polymerase gene expected to lie downstream of the intein gene (i.e. the coding sequence for the C-extein) is missing (additional file 2). Comparisons with the trace archives suggest that these are all genuine mutations. Phylogenetic analyses indicate that this degenerate RNA polymerase gene is not closely related to eukaryotic RNA polymerase I, II or III genes (Figure 2). Instead, it is most closely related (100% bootstrap support) to an RNA polymerase from African swine fever virus (ASFV), a large double-stranded DNA virus that is a member of the nuclear-cytoplasmic large dsDNA virus (NCLDV) group [38]. In addition, three intact genes encoding the second largest subunits of RNA polymerases I, II and III can be found in the *P. ramorum *genome (Figure 2). Close relatives (not shown) of these three genes also appear in the genome sequence of the related species *Phytophthora sojae *(also sequenced by the JGI), but no close relative of the degenerate ASFV-like RNA polymerase gene is present in the *P. sojae *genome.

Further analyses of the sequences surrounding this RNA polymerase gene reveal a likely explanation for its unusual features; when the predicted products of the ORFs in the regions close to the RNA polymerase gene are used in BLASTP searches against the protein sequences in GenBank, the strongest hits are (as with the RNA polymerase itself) often proteins encoded by ASFV (additional files 3 and 4). Most of these proteins do not have close relatives in the *P. sojae *genome. The ORFs further away from the degenerate RNA polymerase gene, however, do have close matches in *P. sojae*, and are not closely related to genes found in ASFV. It is therefore likely that a previously unidentified virus related to ASFV has integrated into the *P. ramorum *genome. This integration would have occurred after the divergence of the lineages leading to *P. ramorum *and *P. sojae*, as no trace of the putative viral relic appears in *P. sojae*. After its integration into the *P. ramorum *genome, the viral sequence has started to degenerate.

### An intein in an RNA polymerase sequence isolated from the Sargasso Sea

A putative intein was also identified in a sequence from an unclassified species (IBEA_CTG_SVAEH23TF) found in the environmental samples division of GenBank (accession no. AACY01369547). The sequence was generated as part of the shotgun sequencing of samples from the Sargasso Sea [39]. The complementary strand of this sequence encodes the N-terminal part of an intein, which includes the conserved splicing motifs and the first motif of a homing endonuclease domain, and is preceded by part of an RNA polymerase. The intein in this sequence is inserted at the same site as that in the putative viral relic in *P. ramorum*, i.e. they are allelic inteins. Similarity searches at InBase indicate that the most closely similar annotated intein to this Sargasso Sea sequence is Ceu ClpP, the intein from the chloroplast of *C. eugametos *(E = 2 × 10^-13^). BLAST2 comparisons suggest a closer sequence similarity between the Ceu ClpP intein and the Sargasso Sea sequence (E = 1 × 10^-17^) than between the Sargasso Sea sequence and the *P. ramorum *virus intein fragment (E = 4 × 10^-5^).

Phylogenetic analyses (not shown) indicate that the RNA polymerase from which this sequence is derived is most closely related to eukaryotic RNA polymerase II, although it is not highly similar to any sequence of known origin. E-values derived from TBLASTN searches at NCBI [40] indicate that the 59-residue fragment of this RNA polymerase is most similar (52–56% amino acid identity) to RNA polymerases (RPB2) from fungi (Table 2). It is less likely that the sequence is from a marine virus such as one of the large double-stranded DNA viruses from the NCLDV group that infect eukaryotes (40–46% amino acid identity). These large viruses, some of which infect marine organisms, encode RNA polymerase II-like proteins.

### An intein in an RNA polymerase sequence found in an isolate of the *Emiliania huxleyi *virus

A partial intein sequence was identified in a short sequence cloned from *E. huxleyi *virus 163 (GenBank accession DQ127798). The allelic site in *E. huxleyi *virus 86 (accession CAI65861, containing sequence annotated as encoding a RPB2 homologue) does not contain an intein. *E. huxleyi *is a marine calcifying haptophyte alga, and the virus is a member of the NCLDV group [41]. The intein-like sequence represents only ~50 residues of the C-terminal end of an intein similar to SasRPB2-b and CstRPB2-b (it ends in TGN). The sequence downstream (the C-extein) from the intein-like sequence in *E. huxleyi *virus 163 encodes residues almost identical to the corresponding region in *E. huxleyi *virus 86 (Figure 1). This region is immediately adjacent the region corresponding to the insertion site of the *P. ramorum *virus partial intein and the partial intein from the Sargasso Sea isolate – that is, these three partial inteins are allelic inteins (Figure 1).

### RNA polymerase inteins insert at highly conserved sites

**Figure 3:**
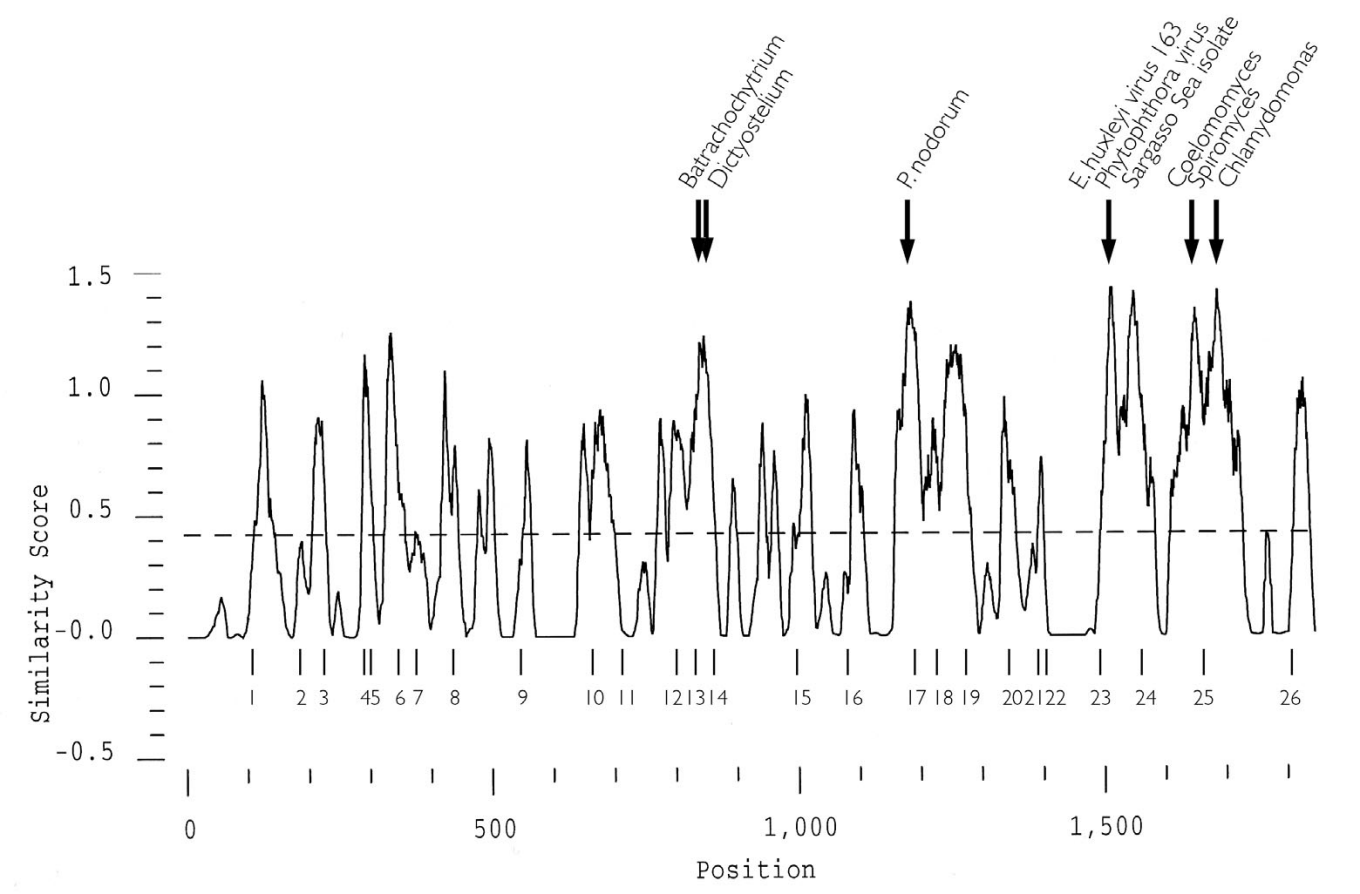
**Profile of RNA polymerase alignment showing high level of conservation at intein insertion sites**. The plot was generated from an alignment of multiple eukaryotic RNA polymerase I, II and III sequences using the PLOTSIMILARITY program of the GCG package of sequence analysis programs [49]. Intein location positions are as follows: BdeRPB2-c 843; Ddi RPC2 853; PnoRPA2 1195; *P. ramorum *virus, Sargasso Sea isolate, *E. huxleyi *virus 163 1516; SasRPB2-b, CstRPB2-b 1664; CreRPB2-a 1696. Intron locations are indicated by a short vertical line at their insertion site and are as follows: *D. discoideum *1–111, 2–184; *P. nodorum *5–301, 17–1192; *B. dendrobatidis *13–826, 21–1357; *C. reinhardtii *3–222, 4–295, 6–335, 7–376, 8–439, 9–545, 10–657, 11–709, 12–797, 14–867, 15–998, 16–1093, 18–1217, 19–1287, 20–1346, 22–1401, 23–1511, 24–1587, 25–1688, 26–1817.

Previous analyses have suggested that inteins usually appear at highly conserved sites within their host proteins [5]. One possible reason for this preference is that inteins inserted at such sites are less likely to be removed. Highly conserved sites in proteins usually have important and sequence-specific functions, and, therefore, any deletion that removes the intein sequence would have to be very precise or it would result in a non-functional host gene. In contrast, inteins inserted at poorly conserved sites might be successfully removed by a wide range of imprecise deletions. A second possibility is that inteins inserted at highly conserved sites may be more likely than inteins inserted at poorly conserved sites to spread successfully throughout the gene pool of a species or to undergo a successful horizontal transmission to a new species, as the homing endonuclease recognition site is more likely to be conserved. With six distinct insertion sites within homologous genes, these new RNA polymerase inteins provide a good opportunity to examine this phenomenon in detail in a eukaryote system. We therefore created an alignment of eukaryotic RNA polymerases, plotted the level of conservation in 10 amino-acid windows across the alignment, and mapped the intein insertion sites onto the plot (Figure 3). As can be seen, the intein insertion sites each correspond to one of the peaks in the sequence conservation plot, indicating that each is inserted into one of the most highly conserved sites within these genes. Not all highly conserved sites can act as intein refuges because they must contain appropriate flanking residues (for example the C-extein Cys or Ser).

We also mapped onto the plot the positions of all the spliceosomal introns from the intein-containing RNA polymerase genes. There are two introns in the genes from *P. nodorum*, *B. dendrobatidis *and *D. discoideum*, and 20 in the *C. reinhardtii *gene. None was found in the *S. aspiralis *or *C. stegomyiae *genes or in the putative proviral gene from *P. ramorum*. As can be seen in Figure 3, some introns are inserted at highly conserved positions, but others are inserted at sites that are only moderately or are poorly conserved, showing that, in contrast to inteins, RNA polymerase introns do not preferentially appear at highly conserved sites.

**Figure 4:**
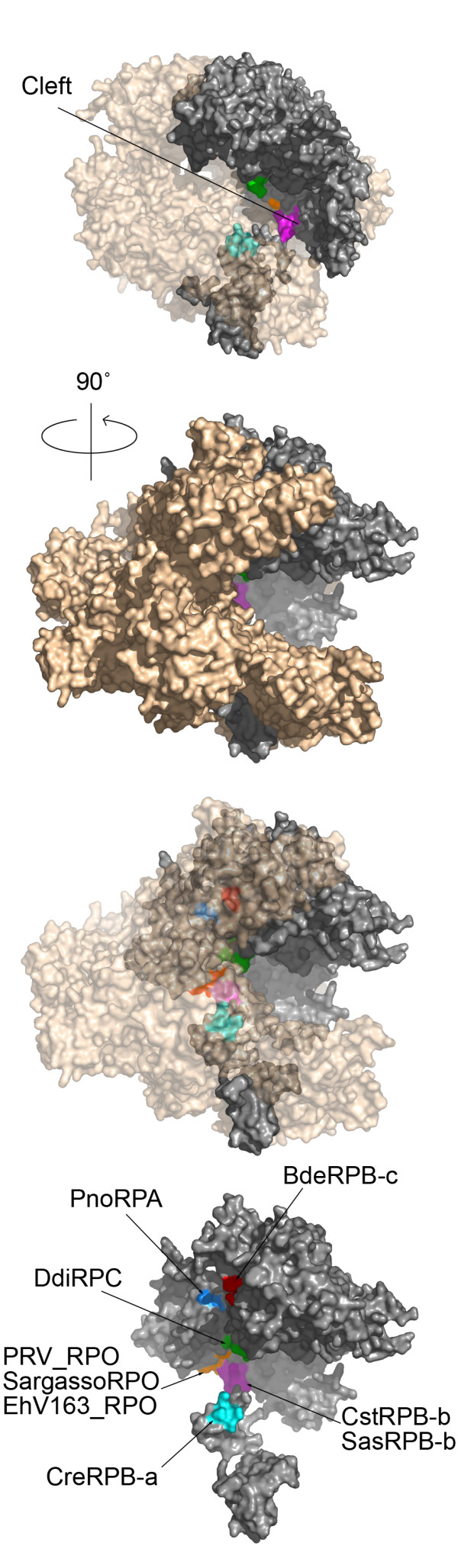
**The position of the six RNA polymerase intein insertion sites mapped onto the crystal structure of the RNA polymerase II of *Saccharomyces cerevisiae***. Representation of *S. cerevisiae *RNA polymerase II (PDB: 1I3Q); the second largest subunit is coloured dark grey (other subunits, including the largest, are coloured light brown). Top: surface view showing the position of the cleft formed by the two largest subunits and the position of the four intein insertion sites (indicated by different colours) on the surface of the cleft, near the active site/"wall" region. Lower: three surface views from a different orientation; the middle image has all of the subunits other than the second largest subunit as a semi-transparent surface so that the position of the two intein insertion sites on the interface between RPB1 and RPB2 (red and blue regions) can be seen. The lowest image is of RPB2 only, but with the intein insertion sites labelled with the names of inteins inserted at these positions in some homologues.

Having mapped the intein insertion sites onto the eukaryotic RNA polymerase sequence conservation plot (Figure 3) and determined that these were in regions of high sequence conservation, we wished to discover where these sites occurred in the three-dimensional protein, including which structural domains correspond to the intein insertion sites. It is of interest to plot these sites because inteins at some positions might be more easily processed during protein folding. Because the RNA polymerase subunits RPA2, RPB2 and RPC2 are similar in structure, we used as our common template the structure of RPB2 from *S. cerevisiae *[42,43]. It is possible to map onto the protein structure the position of the RPA2, RPB2, RPC2 and viral RPO II intein insertion sites, as if they were all inserted into the homologous RPB2 protein. Using data from the Protein Data Bank [44] (entry 1I3Q) and the MacPyMOL molecular visualisation system [45], we highlighted six residues immediately adjacent to each intein insertion site. Figure 4 illustrates the assembly of RPB2 and RPB1 into the core heterodimer. Using the terminology of Cramer *et al *[42], both the Ddi RPC2 and Bde RPB2 insertion sites are in the 'fork' domain, the Pno RPA2, Cst RPB2, Sas RPB2 and the viral RPO intein found in *P. ramorum *are all inserted within the 'hybrid binding' domain, and the Cre RPB2 intein insertion site is in the anchor domain. All of these sites are close to the active site of RPB2. None of the insertion sites are found on the external surface of the protein; all are on the surface of the 'cleft' formed by the RPB1/RPB2 heterodimer or are on the interface between these subunits (Figure 4). It is highly probable, therefore, that inteins inserted into these sites in any of the homologues will need to be accurately spliced out before the protein subunits can assume their correct folds and the active RNA polymerase complex can be assembled. In contrast, if inteins were present on the surface of the heterodimer, they could undergo inactivation and progressive deletion without necessarily impairing the assembly and function of the RNA polymerase.

### Relationships among inteins

Previous work with eukaryotic inteins has shown that allelic inteins are usually each other's closest relatives. For instance, the wide variety of PRP8 inteins identified in ascomycete and basidiomycete fungi form a monophyletic group, relative to all other known inteins. Similarly, the yeast VMA1 inteins also appear as a monophyletic group. There is some evidence to suggest that many of the previously identified eukaryotic nuclear inteins (i.e., VMA1, PRP8, GLT1 and CHS2) may be more closely related to each other than they are to most inteins encoded by non-nuclear genes [20]. There is no evidence suggesting that the nuclear-encoded inteins are closely related to eukaryotic inteins encoded by chloroplast genes, or inteins encoded by eukaryotic viruses. Indeed, some of these latter inteins are alleles of, and closely related to, inteins found in prokaryotes. For instance, the DNA polymerase B inteins of the *A. polyphaga *mimivirus and HaV01 are most closely related to allelic DNA polymerase inteins from various archaea [17].

**Figure 5:**
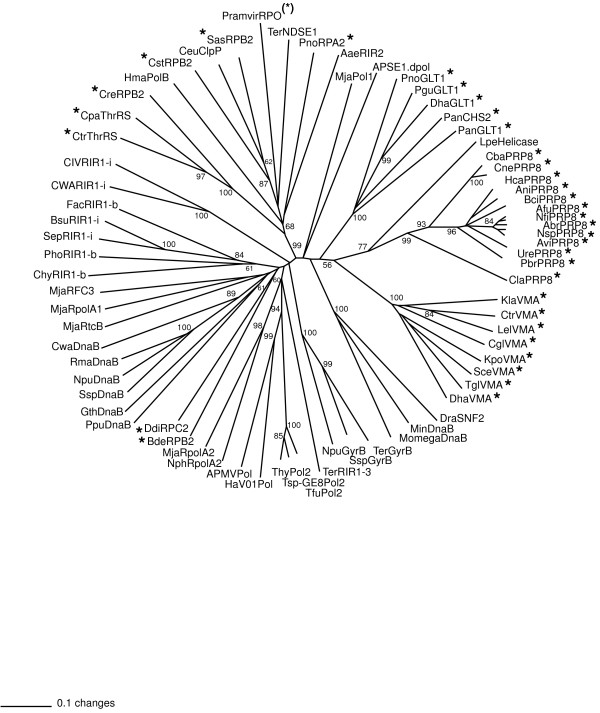
**Phylogenetic tree of intein splicing domains**. This unrooted distance tree was constructed by the neighbour-joining method using PAUP*4b10 [52] with the default settings. Numbers on the branches indicate the percentages of bootstrap support derived from a heuristic search with 100 random addition replicates; this search included a tree-bisection-reconnection branch-swapping algorithm. All bootstrap values > 50 have been reported except in cases where allelic inteins fall within a well-supported (95–100% bootstrap) group, when some values >50 have been omitted for reasons of space. Inteins encoded by nuclear genes are highlighted with an asterisk. The alignment used is available as supplementary data (additional file 5).

To study the relationships among the new RNA polymerase inteins and previously identified inteins, we constructed phylogenies based on alignments of conserved intein splicing domains. Homing endonuclease domains were not included in this analysis because they are absent from mini-inteins and because, even in full-length inteins, they are often degenerate and therefore might produce misleading results. The sequences to be aligned were edited so as to remove all of the intein residues between the end of the N-terminal splicing domain and the beginning of the C-terminal splicing domain. These domains were determined by comparison with other intein sequences available at InBase [6]. The resulting alignment has 102 positions and is available as supplementary data (additional data file 5). An example of a tree containing all the new RNA polymerase inteins and a wide variety of previously identified inteins, including most of the known eukaryotic inteins and representatives of many of the allele groups of prokaryotic inteins, is shown in Figure 5. Most of the relationships observed on this tree are consistent with results obtained previously. For instance, the PRP8 inteins all group together, as do the VMA1 inteins.

The new eukaryotic RNA polymerase inteins do not generally appear to be closely related to each other, despite being present in homologous (in some cases paralogous) genes. This is not perhaps unexpected, however, as most are inserted at different sites in these genes and therefore are not allelic inteins. The *C. reinhardtii *RNA polymerase II intein appears to be most closely related to the threonyl transfer RNA synthetase inteins from *C. tropicalis *and *C. parapsilosis*. This grouping receives a high level of bootstrap support (100%). This is unusual as these inteins are not alleles and are found in different kingdoms. The *P. nodorum *RNA polymerase I intein is not closely related to any other known intein, although it does fall within a moderately supported (68%) group that also includes the *C. eugametos *chloroplast Ceu ClpP intein, Sas RPB2, Cst RPB2, the putative viral intein embedded within the *P. ramorum *genome, and a variety of prokaryotic inteins. All these inteins (including Cre RPB2, Cpa ThrRS and Ctr ThrRS, Pno RPA2, Sas RPB2 and Cst RPB2, and Ceu ClpP), together with a set of prokaryotic inteins, form a well-supported (99%) cluster distinct from all other inteins. The *B. dendrobatidis *RNA polymerase II intein (Bde RPB2) and the *D. discoideum *RNA polymerase III intein (Ddi RPC2) lie outside of this cluster. Although on this tree, they appear as each other's closest known relative, this grouping does not receive high levels of support (60%) and the two inteins are not particularly similar in sequence (~20% identity), so the significance of the grouping is uncertain.

The topology of the distance trees generated from the alignment data is generally very similar if different tree-building algorithms such as quartet puzzling or parsimony analyses are used. The bootstrap values generated follow a similar pattern also, with one exception; the node that joins the PRP8 allelic inteins with the VMA allelic inteins can attract values ranging from 56% to 96%. The bootstrap value of the node that groups many of the newly described RNA polymerase inteins into a cluster distinct from all other inteins ranges from 70% (fast heuristic search, no branch-swapping) to 99% (heuristic search with branch-swapping).

## Discussion

We have identified coding sequences for seven new inteins within nuclear genes. These are all present within homologous genes encoding the second largest subunits of RNA polymerase. One is present in an RNA polymerase I subunit, four (including two allelic inteins) in a RNA polymerase II subunit, one in RNA polymerase III, and the last is found in a viral RNA polymerase in a degenerate provirus. In addition, we identified a sequence from an unknown organism from the Sargasso Sea that contains a partial sequence of an intein allelic to that of the provirus, and a partial sequence of a further allelic intein from *E. huxleyi *virus 163. These new inteins raise the number of distinct (non-allelic) nuclear-encoded inteins identified to 11 (or 10 if the proviral intein is excluded).

The new inteins from *C. reinhardtii*, a green alga, and from *D. discoideum*, a cellular slime mould (Amoebozoa), are the first nuclear-encoded inteins to be found outside of the fungi. These findings indicate that there is no particular barrier to the functioning of inteins in non-fungal eukaryote nuclei. They also have implications for our understanding of the origins and evolution of nuclear inteins. For instance, they suggest either that inteins have a much longer history in nuclear genomes than was previously evident, or perhaps that they have invaded nuclear genomes on multiple occasions or are capable of widespread horizontal transmission. They also suggest that inteins will be identified in further diverse eukaryotes as more genome sequences are determined.

Inteins have now been found in many kingdoms of eukaryotes. They are present in Opisthokonts (in many fungal species and in the viruses of insects), in Amoebozoa (*Dictyostelium *RPC2 and the mimivirus intein, APMV PolB, in *Acanthamoeba*), in green plants (*C. reinhardtii *RPB2 and the *C. eugametos *plastid ClpP protease), in the red alga (the plastid DnaB helicase of *P. purpurea*) and a cryptophyte (the plastid DnaB helicase of *G. theta*). Inteins are found in the viruses of haptophyte algae (*E. huxleyi *virus intein, EhV163_RPO) and viruses of Stramenopiles, both photosynthetic golden-brown algae (*Heterosigma *virus intein, HaV01 PolB), and the non-photosynthetic oomycete (*P. ramorum*, PrV_RPO).

The intein from the viral relic embedded in *P. ramorum *is the first example of an intein in a eukaryotic provirus. This intein is of particular interest in the context of the possibility of horizontal transmission of inteins, as it has been suggested that viruses might mediate the movement of inteins between species [17]. For instance, an intein present in a particular gene in a cellular genome might be able to home to a homologous gene in an infecting virus. If this virus were then to infect a second species, the intein could potentially undergo a second homing reaction and become inserted into the homologous gene in the new species. This idea is supported by the presence of allelic inteins in bacteriophage and bacterial genomes [6]. For example, allelic DnaB-b inteins are found in ~17 species of eubacteria and in a giant phage found in *Pseudomonas aeruginosa*. Allelic inteins in the RIR1-i insertion site of ribonucleoside-diphosphate reductase are present in prophages from two strains of *Bacillus subtilis*, in three eukaryote viruses and in a cyanobacterium [6]. Although no nuclear inteins that are alleles of the *P. ramorum *proviral intein have yet been identified, the finding supports the possibility that such a horizontal transmission might take place in eukaryotes. The Sargasso Sea intein fragment may represent such a nuclear-encoded homologue of the *P. ramorum *proviral intein; alternatively it may be derived from a eukaryotic nucleocytoplasmic large DNA virus (NCLDV). An intein fragment is present in the allelic site of one isolate of the *E. huxleyi *virus, a member of the NCLDV group. However, the *P. ramorum *proviral intein is intriguing, because the chances of a successful intein transmission from virus to host would be increased by the integration of the viral DNA into the host genome, as then the viral DNA would be a stable part of the host genome and would be available to act as a template for DNA repair (an essential part of the homing process) for much longer than in a transient infection.

The six sites where the inteins are inserted are among the most highly conserved regions of the second largest subunit of RNA polymerase. This is consistent with previous findings that inteins are usually found at highly conserved sites. It is not clear why RNA polymerase has so many inteins, however, when no other nuclear gene has more than one known intein. It is possible that it is related to the presence of RNA polymerase genes in a variety of viruses. This may increase the likelihood of an intein being horizontally transferred, which, according to the proposed lifecycle of inteins, may increase the likelihood of it surviving for long periods of time. Multiple alleles were detected at two of the new intein sites; the other four sites were represented by single inteins. This emphasises the extremely sporadic distribution of inteins. Many examples of RNA polymerase genes have been sequenced, because of their usefulness in phylogenetic studies, but inteins have been found in few.

The non-allelic RNA polymerase inteins are not highly similar to each other, or to any previously identified inteins. Five of the inteins, Pno RPA2, Cre RPB2, Cbe RPB2, Sas RPB2 and the intein from the provirus in *P. ramorum*, however, form part of a well-supported but diverse group of inteins that also includes the *Candida *ThrRS inteins, Ceu ClpP and several prokaryotic inteins. Within this group, the Cre RPB2 intein appears to be most closely related to the ThrRS inteins (100% bootstrap support), which is unusual as these are not allelic inteins. Similarly, Cst RPB2 and Sas RPB2 form a well-supported group with the non-allelic Ceu ClpP intein. These findings raise the possibility that, in each of these cases, one of the alleles is derived from the other via the ectopic movement of an ancestral intein. There is, however, no obvious similarity among the nucleotide sequences that flank these non-allelic inteins. Such similarity might have suggested that a homing endonuclease had cleaved a degenerate site and promoted an ectopic conversion, but it is unlikely to be detected; even the allelic inteins CstRPB2 and SasRPB2 show <80% sequence identity in this region (all but two of the changes are third codon substitutions).

The finding that clades representing nuclear-encoded inteins are dispersed throughout the intein phylogeny, intermingled with clades representing eubacterial, archaeal and viral inteins (Figure 5), suggests that inteins have a very long history in eukaryotes, dating back to eukaryotic origins, and/or that horizontal intein transmission between eukaryotes and prokaryotes has occurred at multiple points. Given the lack of compelling evidence for the occurrence of horizontal transmission of eukaryotic inteins (i.e. there are no examples of highly similar inteins in distantly related host species), together with the general high degree of diversity in the intein sequences, we favour the former possibility that inteins were present in the very earliest eukaryotes. Their present-day sporadic distribution is likely to be primarily the result of multiple, independent losses in different lineages.

## Conclusion

Seven complete new nuclear-encoded inteins were identified and characterised. These inteins were all found in genes encoding the second-largest subunits of RNA polymerase. The inteins were found at six distinct (non-allelic) sites, i.e., only two of them are allelic. Four of the inteins are from fungi (one from an ascomycete, one from a zygomycete and two from chytrids). One intein was found in the green alga *C. reinhardtii *and one in the slime mould *D. discoideum*. These are the first nuclear-encoded inteins from outside of the fungi. The seventh new intein is from a provirus embedded within the genome of an oomycete (the kingdom Stramenopiles). These new inteins substantially increase the number of described nuclear-encoded inteins and also widen the diversity of species known to harbour such inteins. The data suggest that inteins have a long history in eukaryotes, probably dating back to their earliest origins.

## Methods

### Sequence databases

The sequence databases used were:

• The Joint Genome Institute [33].

• The Wellcome Trust Sanger Institute [46].

• The Broad Institute [32].

• Washington University Genome Sequencing Center [47].

• National Center for Biotechnology Information [48].

### Bioinformatics analyses

General sequence analyses were carried out using the programs of the GCG package [49]. Sequence similarity searches were carried out using the BLAST servers at GenBank [40], InBase [6] or the various genome-sequencing centres mentioned above. Multiple sequence alignments were constructed using CLUSTAL_X [50] and refined using SEAVIEW [51]. Phylogenetic analyses were performed using PAUP* [52] using the default settings unless otherwise noted.

### Sequences

Intein protein sequences were retrieved from InBase [6] under the standard intein names. Protein sequences for the second largest subunits of RNA polymerase sequences were retrieved from GenBank [48] using the following protein ID numbers.

### Eukaryotes

• *Schizosaccharomyces pombe *Pol. I, CAB66435; Pol. II, Q02061; Pol III, CAA93558.

• *Aspergillus fumigatus *Pol. I, EAL88681; Pol. II, EAL84702; Pol III, EAL87958.

• *Saccharomyces cerevisiae *Pol. I, AAA34993; Pol. II, CAA99357; Pol III, CAA99422.

• *Dictyostelium discoideum *Pol. I, EAL60592; Pol. II, EAL63310; Pol III, EAL63250.

• *Homo sapiens *Pol. I, AAX81999; Pol. II, AAH23503; Pol III, AAH46238.

• *Drosophila melanogaster *Pol. I, AAF51503; Pol. II, AAF55024; Pol III, AAF58590.

• *Arabidopsis thaliana *Pol. I, AAG52049; Pol. II, CAB36815; Pol III, BAB11387.

• *Cryptosporidium parvum *Pol. I, EAK88354; Pol. II, EAK90367; Pol III, EAK87469.

• *Encephalitozoon cuniculi *Pol. I, CAD26190; Pol. II, CAD25744; Pol III, CAD25947.

• The *P. nodorum *RNA polymerase sequences were predicted from the genes on the following sequences: RNA Pol. I, AAGI01000064; RNA Pol. II, AAGI01000234; RNA Pol. III, AAGI01000034.

• The *P. ramorum *RNA polymerase sequences were predicted from the genes on the following sequences: RNA Pol. I, scaffold 163; RNA Pol. II, scaffold 60; RNA Pol. III, scaffold 33.

• The *Chlamydomonas reinhardtii *RNA polymerase II gene sequence was assembled from sequences from version 2 of the genome assembly (Scaffold 5. contigs 26, 27 and 28) combined with sequences from the trace archive (589516860, 651002588, 591226556, 650233847, 587272333). Introns were identified by comparison to other RNA polymerases.

### Eukaryotic viruses

• African swine fever virus AAA65283

• *Emiliana huxleyi *virus 86 CAI65861

• *Acanthamoeba polyphaga *mimivirus AAQ09583

• Chilo iridescent virus AAK82288

• Grouper iridovirus AAV91067

• Frog virus 3 AAT09722

• Lymphocystis disease virus AAU10873

• Rock bream virus AAT71848

• Swinepox virus AAL69852

• Orf virus AAR98326

• Melanoplus sanguinipes entomopoxvirus T28316

• Amsacta moorei entomopoxvirus AAG02772

• Vaccinia virus AAB96526

### Archaea

• *Pyrococcus furiosus *AAL81688

• *Ferroplasma acidiarmanus *EAM93828.

### Bacteria

• *Staphylococcus aureus *AAW37698

• *Crocosphaeria watsonii *EAM50876

## Authors' contributions

TG participated in intein discovery and the initial data analyses. MB participated in the phylogenetic analyses and examination of aspects of RNA polymerase structure. RP contributed to the design of the study and to the analysis of the results. All of the authors participated in the manuscript preparation and have read and approved the final version.

## Supplementary Material

Additional File 1 **An alignment of RNA polymerase sequences**. Taken from accession data as described in the Methods section. Click here for file

Additional File 2 **The nucleotide sequence and three-frame conceptual translation of the putative RNA polymerase from *P. ramorum***. The RNA polymerase protein sequence is shaded in red and the intein sequence in blue. Click here for file

Additional File 3 **The genomic context of the intein-coding sequence in *Phytophthora ramorum***. The diagram depicts the structures of contigs 4, 5 and 6 of scaffold 19 of the assembled *P. ramorum *genome sequence. ORFs are represented by the shaded boxes. Blue-shaded boxes represent ORFs having a high quality match (E<1 × 10^-30^) in the assembled *Phytophthora sojae *genome sequence (see additional file 4). Red-shaded boxes represent ORFs whose best matches among all the protein sequences in GenBank are proteins coded by African swine-fever virus (additional file 4). The intein and associated RNA polymerase are represented by ORFs 6, 7 and 8 of contig 5. ORFs are as determined by the ORF finder program , except ORF1 of contig 4, which was extended back to the previous stop codon. Contig 6 extends further than the sequence depicted here. Click here for file

Additional File 4 Matches to the ORFs in *P. ramorum *scaffold 19, contigs 4, 5 and 6. Click here for file

Additional File 5 An alignment of the intein splicing domains used to create Figure 5. Click here for file

## Figures and Tables

**Table 1:** Newly described inteins from the second largest subunit of RNA polymerases.

Intein	Organism	Taxonomic group	Allele	size
Pno RPA2	*Phaeosphaeria nodorum*	Ascomycota	RPA2-a	456
Cre RPB2	*Chlamydomonas reinhardtii*	Green alga	RPB2-a	431
Cst RPB2	*Coelomomyces stegomyiae*	Chytrid	RPB2-b	362
Sas RPB2	*Spiromyces aspiralis*	Zygomycota	RPB2-b	354
Bde RPB2	*Batrachochytrium dendrobatidis*	Chytrid	RPB2-c	488
Ddi RPC2	*Dictyostelium discoideum*	Amoebozoa	RPC2-a	464
PrV RPO	*Phytophthora ramorum *virus	StramenopileNCLDV?	RPO-a	incomplete
Unnamed	Unclassified Sargasso sea	unknown	RPO-a	incomplete
EhV RPO	*Emiliana huxleyi *virus 163 *	Haptophyte NCLDV	RPO-a	incomplete

*No intein is present at the allelic site in another *Emiliana huxleyi *virus isolate, *Emiliana huxleyi *virus 86.

Intein size is expressed as amino-acid residue number. NCLDV indicates a member (or putative member) of the nucleocytoplasmic large DNA virus group.

**Table 2:** The unclassified sequence from the Sargasso Sea is unlikely to represent a fragment of a viral genome. TBLASTN searches were conducted at NCBI using as a query the 59 residues from the Sargasso Sea sequence (Accession AACY01369547) that formed the putative C-extein. These 59 residues are encoded on the complementary strand, from base 556 to base 732. Each search was restricted to one of the six groups outlined below.

Accession	Sequences producing significant alignments	E value
Fungi		
gb DQ302778.1	*Entomophthora muscae *AFTOL-ID28, RPB2	4 × 10^-9^
gb DQ521419.1	*Auricularia auricula-judae *AFTOL-ID1681	7 × 10^-9^
gb DQ234553.1	*Hydnum albomagnum *AFTOL-ID 471, RPB2	1 × 10^-8^
gb AY485624.1	*Hydnum repandum *RPB2	1 × 10^-8^
gb DQ302787.1	*Umbelopsis ramanniana *AFTOL-ID 144, RPB2	2 × 10^-8^
Metazoa		
gb XM_793194.1	*Strongylocentrotus purpuratus *LOC593725	5 × 10^-7^
dbj AK114672.1	*Ciona intestinalis *cDNA, clone:cieg010h22	1 × 10^-6^
gb AC007441.9	*Drosophila melanogaster *clone BACR10E03	2 × 10^-6^
gb BT028050.1	*Gasterosteus aculeatus *clone CNB114-G10	2 × 10^-6^
gb U10333.1	*Caenorhabditis elegans *RNA polymerase II	2 × 10^-6^
Plantae		
emb AJ565937.1	MGU565937 *Mimulus guttatus *partial RPB2	9 × 10^-7^
gb DQ029103.1	*Spirogyra sp*. UWCC FW670 RPB2	1 × 10^-6^
gb AF020844.1	*Marchantia polymorpha *RPB140 (RPB2)	1 × 10^-6^
gb AY596718.1	*Tetralocularia pennelii *(RPB2)	1 × 10^-6^
gb AY563264.1	*Selaginella densa *RNA polymerase II (RPB2)	1 × 10^-6^
emb AJ566358.1	GSP566358 *Gardenia sp*. Oxelman 2319 (RPB2)	1 × 10^-6^
Archaea		
gb AE010299.1	*Methanosarcina acetivorans *str. C2A (rpoB)	2 × 10^-6^
gb AE008384.1	*Methanosarcina mazei *strain Goe1 (rpoB)	2 × 10^-6^
gb CP000099.1	*Methanosarcina barkeri *str. fusaro	3 × 10^-6^
gb AE000782.1	*Archaeoglobus fulgidus *DSM 4304 (rpoB1)	4 × 10^-6^
emb BX957222.1	*Methanococcus maripaludis *S2	5 × 10^-6^
emb X14818.1	*Sulfolobus acidocaldarius *rpoB	9 × 10^-6^
Viruses		
gb AF389451.1	Tiger frog virus, complete genome	2 × 10^-4^
gb AY548484.1	Frog virus 3, complete genome	4 × 10^-4^
gb AY666015.1	Grouper iridovirus, complete genome	0.001
gb AY150217.1	*Ambystoma tigrinum stebbensi *virus	0.001
gb AF397202.1	*Regina ranavirus *clone PstI-3.8	0.001
gb AY653733.1	*Acanthamoeba polyphaga *Mimivirus	0.001
emb AJ890364.1	*Emiliania huxleyi *virus 86 isolate Ehv86	0.003
Eubacteria		
	No significant similarity found.	
