# Supplementary material for: Multiple, non-allelic, intein-coding sequences in eukaryotic RNA polymerase genes
Source: BMC Biol. 2006 Oct 27;4:38. doi: 10.1186/1741-7007-4-38 (PMC1635734; doi:10.1186/1741-7007-4-38)
Supplement: Additional File 2 — The nucleotide sequence and three-frame conceptual translation of the putative RNA polymerase from P. ramorum. The RNA polymerase protein sequence is shaded in red and the intein sequence in blue. [file 1741-7007-4-38-S2.doc]

. . . . . .

1 TTAATATACCCACAAGCGTGCGCAGGTTACACTTTCAAAACTCTTCACTCCCCGTCAACT 60

L I Y P Q A C A G Y T F K T L H S P S T

* Y T H K R A Q V T L S K L F T P R Q L

N I P T S V R R L H F Q N S S L P V N Y

. . . . . .

61 ATGTCGTTCACTCTTGATCAAGAACAGTTGATTCCTATCATTGAGTCTGCCCTTACCAAG 120

M S F T L D Q E Q L I P I I E S A L T K

C R S L L I K N S * F L S L S L P L P R

V V H S * S R T V D S Y H * V C P Y Q G

. . . . . .

121 GACAGTGTCATTGGAGCCACGCTTAAGTCGTTTGATCATTTCGTTGAAAATGGTATTCGA 180

D S V I G A T L K S F D H F V E N G I R

T V S L E P R L S R L I I S L K M V F D

Q C H W S H A * V V * S F R * K W Y S T

. . . . . .

181 CAGATCGCTACACAAGTGTTTGACTTGCGTTTCGATCTCGATGCTAAGGATACGGTGGAG 240

Q I A T Q V F D L R F D L D A K D T V E

R S L H K C L T C V S I S M L R I R W S

D R Y T S V * L A F R S R C * G Y G G A

. . . . . .

241 CGCGATGGAACTATTGAGAAGTACTCTCTTGAGATCACGATCGATGATGTTCGCATCAGC 300

R D G T I E K Y S L E I T I D D V R I S

A M E L L R S T L L R S R S M M F A S A

R W N Y * E V L S * D H D R * C S H Q Q

. . . . . .

301 AATCCTTCCAAGTATGATCCTTCCACGCAACACAACTCTCCAATGTTCCCGAATAAAGCT 360

N P S K Y D P S T Q H N S P M F P N K A

I L P S M I L P R N T T L Q C S R I K L

S F Q V * S F H A T Q L S N V P E * S F

. . . . . .

361 TTGTTGAAAGACCTTACGTATTGCTCCGGCGTGTACGTTGATGTTACTATTGTCGCCAAG 420

L L K D L T Y C S G V Y V D V T I V A K

C * K T L R I A P A C T L M L L L S P R

V E R P Y V L L R R V R * C Y Y C R Q G

. . . . . .

421 GCGTTTCATTCTAATGAAACCACCAGCGTCGAGAAGAGTAACATTGACAACACAATGATA 480

A F H S N E T T S V E K S N I D N T M I

R F I L M K P P A S R R V T L T T Q * Y

V S F * * N H Q R R E E * H * Q H N D M

. . . . . .

481 TGCAAGCTACCGACCATGGTCAAGAGTAAGATGTGCAACACGTACAACCGCTCGAACGAA 540

C K L P T M V K S K M C N T Y N R S N E

A S Y R P W S R V R C A T R T T A R T K

Q A T D H G Q E * D V Q H V Q P L E R S

. . . . . .

541 GCGCTGATTCATCTCCAAGAAGATCCTTCTGATCTTGGCGGATACTGTGTGATCAAAGGC 600

A L I H L Q E D P S D L G G Y C V I K G

R * F I S K K I L L I L A D T V * S K A

A D S S P R R S F * S W R I L C D Q R Q

. . . . . .

601 AATCGGTACATCATCATCAATAAGGAAAGCATGAAGTACAACGAAAGCCGCGAGTTTGCG 660

N R Y I I I N K E S M K Y N E S R E F A

I G T S S S I R K A * S T T K A A S L R

S V H H H Q * G K H E V Q R K P R V C E

. . . . . .

661 AACGAAGGACATAAAAATGAACGTTGCCGCGCTGACATCATCAGCAAACCAGGCGACGCT 720

N E G H K N E R C R A D I I S K P G D A

T K D I K M N V A A L T S S A N Q A T L

R R T * K * T L P R * H H Q Q T R R R F

. . . . . .

721 TTTGAAAACTCTTTCAATTGTGTACTCAAGCTGTTGACAAACAATTCAATTGTTATCAAT 780

F E N S F N C V L K L L T N N S I V I N

L K T L S I V Y S S C * Q T I Q L L S I

* K L F Q L C T Q A V D K Q F N C Y Q Y

. . . . . .

781 ATGAGTATGGCTGGTTTCAAAGATATTGAAGTTCCATTCTTCATGTTCTTCCGCGCGCTC 840

M S M A G F K D I E V P F F M F F R A L

* V W L V S K I L K F H S S C S S A R S

E Y G W F Q R Y * S S I L H V L P R A R

. . . . . .

841 GGTGTTTACTCTTCAAAGGATATCATCTCGTACATCACGTATTCGTTCAATGATGCAGAG 900

G V Y S S K D I I S Y I T Y S F N D A E

V F T L Q R I S S R T S R I R S M M Q S

C L L F K G Y H L V H H V F V Q * C R A

. . . . . .

901 CCGATCACGCAGCGCATGCTGAACATCCTGGAACAAGCATTGACGAACACGTACGCAGAC 960

P I T Q R M L N I L E Q A L T N T Y A D

R S R S A C * T S W N K H * R T R T Q T

D H A A H A E H P G T S I D E H V R R H

. . . . . .

961 ATGAATGCTATTCTCAAGAATGAGTTTCCGCACCGCAAGGGAGAGCCTGCAGGATCAGTC 1020

M N A I L K N E F P H R K G E P A G S V

* M L F S R M S F R T A R E S L Q D Q S

E C Y S Q E * V S A P Q G R A C R I S Q

. . . . . .

1021 AACGTCATCACTAACCAGGACGACACCTTGCGTATCTTGACGCGATGCAGTAAGTACTTT 1080

N V I T N Q D D T L R I L T R C S K Y F

T S S L T R T T P C V S * R D A V S T L

R H H * P G R H L A Y L D A M Q * V L *

. . . . . .

1081 GACTCGTACAGATCTAAGTTACGTGCCGCCGGAGAAGAAGACGCATTGAACATTGAACGC 1140

D S Y R S K L R A A G E E D A L N I E R

T R T D L S Y V P P E K K T H * T L N A

L V Q I * V T C R R R R R R I E H * T L

. . . . . .

1141 TTCTTGCTCAGCAAGCTGGAACAGAACATTGATCAGAAGTTCTTGCCGCACATTGGGCTT 1200

F L L S K L E Q N I D Q K F L P H I G L

S C S A S W N R T L I R S S C R T L G L

L A Q Q A G T E H * S E V L A A H W A Y

. . . . . .

1201 ACCCCCGCGGACCGCAAGAAGAAGGCGGCGTACCTTGGTCACATGATTCATAGAATGCTT 1260

T P A D R K K K A A Y L G H M I H R M L

P P R T A R R R R R T L V T * F I E C F

P R G P Q E E G G V P W S H D S * N A S

. . . . . .

1261 CTTGTCAACCTTGGCGTGTTGCAACCAACTGATCGAGACTCGTACAAGAACAAACGCATC 1320

L V N L G V L Q P T D R D S Y K N K R I

L S T L A C C N Q L I E T R T R T N A S

C Q P W R V A T N * S R L V Q E Q T H Q

. . . . . .

1321 AATGATGCAGGCATGTCATACTCGCGAGTCTTCAAGACACAGTTCAACTTCATGGTGGTC 1380

N D A G M S Y S R V F K T Q F N F M V V

M M Q A C H T R E S S R H S S T S W W S

* C R H V I L A S L Q D T V Q L H G G H

. . . . . .

1381 ATGAAGCTGAAGCGCCAGTACATGAAAGACTTCAAGGACAATAGTTTCTCTGACATCAAT 1440

M K L K R Q Y M K D F K D N S F S D I N

* S * S A S T * K T S R T I V S L T S I

E A E A P V H E R L Q G Q * F L * H Q S

. . . . . .

1441 CTAATGGCGCTTTTCAAAAGTGCCATCAAGGCAGATGACTTCGAGAAGGCGCTGATGAAC 1500

L M A L F K S A I K A D D F E K A L M N

* W R F S K V P S R Q M T S R R R * * T

N G A F Q K C H Q G R * L R E G A D E R

. . . . . .

1501 GCAATCGTGAGTGGAGACAAGACATTGACTGTTAATAAGTTAACATTCAAGAATCGACTC 1560

A I V S G D K T L T V N K L T F K N R L

Q S * V E T R H * L L I S * H S R I D S

N R E W R Q D I D C * * V N I Q E S T L

. . . . . .

1561 TCGTCACAACAGCTGCACCATAAGAACAAGCTGAATGTACTAACCACGCTTCGCAGCATT 1620

S S Q Q L H H K N K L N V L T T L R S I

R H N S C T I R T S * M Y * P R F A A L

V T T A A P * E Q A E C T N H A S Q H *

. . . . . .

1621 GACACTCCGGACAAGGGCAACAGCGTCAAATCATCTGAGCGTGCTATCTTGCTACGGCAG 1680

D T P D K G N S V K S S E R A I L L R Q

T L R T R A T A S N H L S V L S C Y G R

H S G Q G Q Q R Q I I * A C Y L A T A G

. . . . . .

1681 GTACATCCGACCGGCACAGGATACATCTGTGGTATCACGTCAGCAGACACTGGTGCGAAG 1740

V H P T G T G Y I C G I T S A D T G A K

Y I R P A Q D T S V V S R Q Q T L V R R

T S D R H R I H L W Y H V S R H W C E G

. . . . . .

1741 GTTGGCATGAGCAAGCAGCTAAGCATCTCCGCTGATATCACTGCAGCATCTTCATCTGAA 1800

V G M S K Q L S I S A D I T A A S S S E

L A * A S S * A S P L I S L Q H L H L K

W H E Q A A K H L R * Y H C S I F I * S

. . . . . .

1801 GTGTTGAAACATATCATTCTTGAAGATGAAGATCTCATTCAGATTCAAATCATTCCTAAG 1860

V L K H I I L E D E D L I Q I Q I I P K

C * N I S F L K M K I S F R F K S F L R

V E T Y H S * R * R S H S D S N H S * G

. . . . . .

1861 GGCATGACGCTACTCACGAAGCATAATCTTCACAAGGTGTTCGTGAACGGTGACTGGCTA 1920

G M T L L T K H N L H K V F V N G D W L

A * R Y S R S I I F T R C S * T V T G *

H D A T H E A * S S Q G V R E R * L A R

. . . . . .

1921 GGATGCGTGCAAGACTTCGCAAGCTTCTTAATGCGATACCGCTTGAAGCGCCGCGAGGGC 1980

G C V Q D F A S F L M R Y R L K R R E G

D A C K T S Q A S * C D T A * S A A R A

M R A R L R K L L N A I P L E A P R G R

. . . . . .

1981 GAGATCAATATGTTTACTACGGTGTCGCACAACATCATTGCCAATGAGATTCGTCTCTGG 2040

E I N M F T T V S H N I I A N E I R L W

R S I C L L R C R T T S L P M R F V S G

D Q Y V Y Y G V A Q H H C Q * D S S L G

. . . . . .

2041 GTTGACTCCTGGCGATTGATCCGTCCGCTACTCATCGTACGAAACAACATTGGCGAGAGT 2100

V D S W R L I R P L L I V R N N I G E S

L T P G D * S V R Y S S Y E T T L A R V

* L L A I D P S A T H R T K Q H W R E W

. . . . . .

2101 GGATACACGCATACCAAGTTCCGCCAATGGATTGACTTCACTGATGATCATGTGAAGAAG 2160

G Y T H T K F R Q W I D F T D D H V K K

D T R I P S S A N G L T S L M I M * R S

I H A Y Q V P P M D * L H * * S C E E A

. . . . . .

2161 CTACAGACGGGTGCTATTGACATTGATGATCTAGCTACTGATGGTACTTATCACCAGAGG 2220

L Q T G A I D I D D L A T D G T Y H Q R

Y R R V L L T L M I * L L M V L I T R G

T D G C Y * H * * S S Y * W Y L S P E E

. . . . . .

2221 AGCACGAGAACACATATATTGCATTCGAGCATGATCGCTTCAAGCAACACCTTACCAACC 2280

S T R T H I L H S S M I A S S N T L P T

A R E H I Y C I R A * S L Q A T P Y Q P

H E N T Y I A F E H D R F K Q H L T N P

. . . . . .

2281 CACTGCATCGCTACTCGCATGTCGACATCCCGCAAGGGAACATGGGACTGGTTGCACTGA 2340

H C I A T R M S T S R K G T W D W L H *

T A S L L A C R H P A R E H G T G C T D

L H R Y S H V D I P Q G N M G L V A L T

. . . . . .

2341 CAAGCGTCTTCGCGAATCACAATCAGGCTGCACGTATTGTGTTCCAGACGAATCAAGTCA 2400

Q A S S R I T I R L H V L C S R R I K S

K R L R E S Q S G C T Y C V P D E S S Q

S V F A N H N Q A A R I V F Q T N Q V K

. . . . . .

2401 AACAGACTAACAGCTGGGCACTCAAGAACTGGGCGTTCGCCGCGCACAAGGATCTCTATC 2460

N R L T A G H S R T G R S P R T R I S I

T D * Q L G T Q E L G V R R A Q G S L S

Q T N S W A L K N W A F A A H K D L Y H

. . . . . .

2461 ATCAAGTATATGTTGAGGATCCTCTTATGAGTACATTCGCGTATCGCCACATCCCGCCGA 2520

I K Y M L R I L L * V H S R I A T S R R

S S I C * G S S Y E Y I R V S P H P A D

Q V Y V E D P L M S T F A Y R H I P P M

. . . . . .

2521 TGTGTACCAACGTGATTGTTGCAATCTCAATCTACGGAGGCTTCAACCAAGAAGACTCTT 2580

C V P T * L L Q S Q S T E A S T K K T L

V Y Q R D C C N L N L R R L Q P R R L F

C T N V I V A I S I Y G G F N Q E D S L

. . . . . .

2581 TGATCGTGAACAAGTCTTCTGTTGATAGAGGACTCTTCGACGCAGCGCATCTGACGTATG 2640

* S * T S L L L I E D S S T Q R I * R M

D R E Q V F C * * R T L R R S A S D V *

I V N K S S V D R G L F D A A H L T Y D

. . . . . .

2641 ACAAGTGTGATATTGAACAAAACGAAATCATCTGTCGTCCCGATCCAAGCAACACTGCTG 2700

T S V I L N K T K S S V V P I Q A T L L

Q V * Y * T K R N H L S S R S K Q H C *

K C D I E Q N E I I C R P D P S N T A D

. . . . . .

2701 ACATCAAGAGTTACAGTAACTACGAGAAGCTCGTGAATGGACTCATCCAGGAAGGAACGT 2760

T S R V T V T T R S S * M D S S R K E R

H Q E L Q * L R E A R E W T H P G R N V

I K S Y S N Y E K L V N G L I Q E G T Y

. . . . . .

2761 ATGTTCAAGAAGGCGACGGCTTAGTAGGCAAAGTAGCAAAGCTACAGAAGGGAGATATGA 2820

M F K K A T A * * A K * Q S Y R R E I *

C S R R R R L S R Q S S K A T E G R Y E

V Q E G D G L V G K V A K L Q K G D M K

. . . . . .

2821 AAGATCCCAATGTGATCTACTCTAACCGTAGCATGGTGTACCGACACAAGGAACCAGCTT 2880

K I P M * S T L T V A W C T D T R N Q L

R S Q C D L L * P * H G V P T Q G T S L

D P N V I Y S N R S M V Y R H K E P A Y

. . . . . .

2881 ACATCTGGAGAGTGATCCACACGCTGAACCATGATGATCACGAGATGGTCAAGATTGTGT 2940

T S G E * S T R * T M M I T R W S R L C

H L E S D P H A E P * * S R D G Q D C V

I W R V I H T L N H D D H E M V K I V F

. . . . . .

2941 TCCAAACGTTCAGAAGCATTTAACTAGGATGTATGTTCTGCTTGACGCCTAAGCATGAGG 3000

S K R S E A F N * D V C S A * R L S M R

P N V Q K H L T R M Y V L L D A * A * G

Q T F R S I * L G C M F C L T P K H E V

. . . . . .

3001 TGCTCACTACTAGAGGATGGATCCCTATTGCTAAGATGACTAAGCAACACAAGATCGCAA 3060

C S L L E D G S L L L R * L S N T R S Q

A H Y * R M D P Y C * D D * A T Q D R N

L T T R G W I P I A K M T K Q H K I A T

. . . . . .

3061 CTATGAGTCCGAAAGGAAAGTTCCGATATGAACAACCAAGTGAGGTAAATGTCTTTGATT 3120

L * V R K E S S D M N N Q V R * M S L I

Y E S E R K V P I * T T K * G K C L * L

M S P K G K F R Y E Q P S E V N V F D Y

. . . . . .

3121 ACCAAGGAAATATTTACAAAGTGTCAAATGCTGCGGTTGATGCGAGACTACAAGATGTAT 3180

T K E I F T K C Q M L R L M R D Y K M Y

P R K Y L Q S V K C C G * C E T T R C M

Q G N I Y K V S N A A V D A R L Q D V C

. . . . . .

3181 GCCAAGATTGAAGACACAGACGAGTTTGAGTTGATCGAAGCACAAGACCTTACTGATCAT 3240

A K I E D T D E F E L I E A Q D L T D H

P R L K T Q T S L S * S K H K T L L I M

Q D * R H R R V * V D R S T R P Y * S *

. . . . . .

3241 GATTATCAAGTAAAGAACTCTATGCCACCACCAACGATCGCTATCCAGCCTACGTTCACC 3300

D Y Q V K N S M P P P T I A I Q P T F T

I I K * R T L C H H Q R S L S S L R S P

L S S K E L Y A T T N D R Y P A Y V H L

. . . . . .

3301 TTACCAACAGGAGAAGAAGTCAAATGAATGATTGGCTTCTGTTCCTCGGTATCTACCTTT 3360

L P T G E E V K * M I G F C S S V S T F

Y Q Q E K K S N E * L A S V P R Y L P L

T N R R R S Q M N D W L L F L G I Y L C

. . . . . .

3361 GCGAAGGACATGTCGACAAAGGCATCATGATCCGCATCAACGCCCACAAGGAACGAGTTA 3420

A K D M S T K A S * S A S T P T R N E L

R R T C R Q R H H D P H Q R P Q G T S Y

E G H V D K G I M I R I N A H K E R V M

. . . . . .

3421 TGACTGCTCTGATAGATGTACTTCCAAAGCTGCACCAAGAGTATAATATCTATCCTGGTA 3480

* L L * * M Y F Q S C T K S I I S I L V

D C S D R C T S K A A P R V * Y L S W Y

T A L I D V L P K L H Q E Y N I Y P G T

. . . . . .

3481 CGCCTAACGATGTGTATTTACTAAGCAAAGGACAGTTCCTAGCTAGGTCGCTCATGCCGC 3540

R L T M C I Y * A K D S S * L G R S C R

A * R C V F T K Q R T V P S * V A H A A

P N D V Y L L S K G Q F L A R S L M P L

. . . . . .

3541 TTGGCAAGGGAGTAGAGAAACGATTTCCTGATTATGTCTGGAGCATGAATGCGCGCCAGT 3600

L A R E * R N D F L I M S G A * M R A S

W Q G S R E T I S * L C L E H E C A P V

G K G V E K R F P D Y V W S M N A R Q S

. . . . . .

3601 CTAGGATCTTGCTTGACGCAATGGTACTTGGAGATGGAGACTATCGTAAAGGATACAATG 3660

L G S C L T Q W Y L E M E T I V K D T M

* D L A * R N G T W R W R L S * R I Q W

R I L L D A M V L G D G D Y R K G Y N G

. . . . . .

3661 GGTTCTACTTCTCATCAGGGTCAATTGATCTAGCCGATGGAGTACAGCGACTTGCACTTC 3720

G S T S H Q G Q L I * P M E Y S D L H F

V L L L I R V N * S S R W S T A T C T S

F Y F S S G S I D L A D G V Q R L A L H

. . . . . .

3721 ACTCAGGACTTGGGTCGCGAGTAGAGATCAAACGCCCCGCTGGCGAAGTACTGAAGATCA 3780

T Q D L G R E * R S N A P L A K Y * R S

L R T W V A S R D Q T P R W R S T E D Q

S G L G S R V E I K R P A G E V L K I K

. . . . . .

3781 AAGGAGTAGAGACTGTTCGCAAGACGAATGCTTATCGAGTGTCGTTCTACTGCAATCAGA 3840

K E * R L F A R R M L I E C R S T A I R

R S R D C S Q D E C L S S V V L L Q S D

G V E T V R K T N A Y R V S F Y C N Q T

. . . . . .

3841 CAACTCAAGCACCATGGATCCGAACCAAACACGCGAAGTTGGTAGAGTATGATGGCAAGG 3900

Q L K H H G S E P N T R S W * S M M A R

N S S T M D P N Q T R E V G R V * W Q G

T Q A P W I R T K H A K L V E Y D G K V

. . . . . .

3901 TCTATTGCCCTACTGTCAAATCTGGTGTCTTTCTCACCAGACTCAACGGAAAGGATCATT 3960

S I A L L S N L V S F S P D S T E R I I

L L P Y C Q I W C L S H Q T Q R K G S L

Y C P T V K S G V F L T R L N G K D H W

. . . . . .

3961 GGACGTAGCGCAAGTCAACTCAATCATGATACATTTGCGTTTCGCGGGCGGGTACTACAA 4020

G R S A S Q L N H D T F A F R G R V L Q

D V A Q V N S I M I H L R F A G G Y Y

T * R K S T Q S * Y I C V S R A G T T
