# Supplementary material for: Multiple, non-allelic, intein-coding sequences in eukaryotic RNA polymerase genes
Source: BMC Biol. 2006 Oct 27;4:38. doi: 10.1186/1741-7007-4-38 (PMC1635734; doi:10.1186/1741-7007-4-38)
Supplement: Additional File 4 — Matches to the ORFs in P. ramorum scaffold 19, contigs 4, 5 and 6. [file 1741-7007-4-38-S4.doc]

**Supplementary file 4. Matches to the ORFs in *P. ramorum* scaffold 19, contigs 4, 5 and 6.**

| ORF^1^ | Coordinates^2^ | Best match in Genbank^3^ | Best match in P. sojae^4^ |
| --- | --- | --- | --- |
| contig4.ORF1 | 1-595 (-3) | XP_001090730, PREDICTED: apolipoprotein A-IV [Macaca mulatta]; E=0.26 | Scaffold 43 (243712-244239). E=6.0e-42 |
|  |  |  |  |
| contig5.ORF1 | 360-1376 (-3) | AAF45927, CG15570-PA [Drosophila melanogaster]; E=8e-07 | Scaffold 2 (730817-840633). E=1.7e-11 |
| contig5.ORF2 | 1787-2719 (-1) | XP_767441, hypothetical protein GLP_238_20700_23057 [Giardia lamblia ATCC 50803]; E=4e-04 | Scaffold 133 (80035-80865). E=2.9e-06 |
| contig5.ORF3 | 3960-4667 (-3) | No match | No match |
| contig5.ORF4 | 4903-5511 (-2) | No match | No match |
| contig5.ORF5 | 6187-6795 (+1) | NP_767215, hypothetical protein bll0575 [Bradyrhizobium japonicum USDA 110]; E=2e-07 | No match |
| contig5.ORF6 | 7915-8559 (-2) | ZP_01392482, MCM [Methanoculleus marisnigri JR1]; E=2e-15 | No match |
| contig5.ORF7 | 8920-9561 (-2) | CAA79698, putative RNA polymerase second largest subunit [African swine fever virus]; E=7e-35 | Scaffold 82 (206754-207098). E=4.0e-09 |
| contig5.ORF8 | 9543-11822 (-3) | CAA79698, putative RNA polymerase second largest subunit [African swine fever virus]; E=2e-98 | Scaffold 82 (207510-209105). E-6.9e-21 |
| contig5.ORF9 | 12044-12634 (-1) | YP_437658, Kef-type K+ transport system, predicted NAD-binding component [Hahella chejuensis KCTC 2396], E=0.15 | No match |
| contig5.ORF10 | 13770-14879 (-3) | CAA50831, helicase [African swine fever virus], E=2e-21 | No match |
| contig5.ORF11 | 15345-17291 (-3) | No match | No match |
| contig5.ORF12 | 17603-18730 (-1) | No match | No match |
| contig5.ORF13 | 19664-21178 (+2) | NP_042761, pC475L [African swine fever virus]; E=1e-14 | No match |
| contig5.ORF14 | 21561-22838 (-3) | AAA42719, LMW6DL [African swine fever virus]; E=0.61 | No match |
| contig5.ORF15 | 22965-23390 (-3) | NP_042794, guanylyltransferase [African swine fever virus]; E=3e-11 | No match |
| contig5.ORF16 | 23545-24747 (-2) | CAA50806, capping enzyme large subunit [African swine fever virus]; E=3e-30 | No match |
| contig5.ORF17 | 24838-25554 (-2) | No match | No match |
| contig5.ORF18 | 26016-26681 (-3) | XP_421895, PREDICTED: similar to Disco-interacting protein 2 homolog [Gallus gallus]; E=0.12 | No match |
| contig5.ORF19 | 27186-27836 (+3) | CAG59636, unnamed protein product [Candida glabrata CBS138]; E=0.86 | No match |
| contig5.ORF20 | 27902-30061 (+2) | NP_042754, pM1249L [African swine fever virus] E=0.022 | No match |
|  |  |  |  |
| contig6.ORF1 | 156-809 (+3) | No match | No match |
| contig6.ORF2 | 2996-3646 (+2) | No match | No match |
| contig6.ORF3 | 4405-5670 (-2) | NP_042793, DNA ligase [African swine fever virus]; E=8e-34 | No match |
| contig6.ORF4 | 5725-6321 (-2) | No match | No match |
| contig6.ORF5 | 8570-13672 (+2) | EAR82081, hypothetical protein TTHERM_01326800 [Tetrahymena thermophila SB210]; E=2e-26 | Scaffold 22 (588162-617852). E=0.0 |
| contig6.ORF6 | 15453-18410 (-3) | ZP_01272258, Exodeoxyribonuclease VII [Psychrobacter sp. Prwf-1]; E=0.36 | Scaffold 3 (486474-489422). E=0.0 |
| contig6.ORF7 | 18598-20655 (-2) | XP_655361, Rho guanine nucleotide exchange factor [Entamoeba histolytica HM-1:IMSS]; E=5e-10 | Scaffold 3 (465837-467711). E=0.0 |
| contig6.ORF8 | 21275-23110 (-1) | No match | Scaffold 3 (431404-470300). E=0.0 |
| contig6.ORF9 | 23930-26320 (+2) | AAF29388, Contains similarity to a vacuolar sorting receptor homolog from  Arabidopsis thaliana gb|U79959 [Arabidopsis thaliana]; E=2e-35 | Scaffold 3 (471364-473694). E=0.0 |

^1^ ORF designations are as in Supplementary Figure 1.

^2^ Coordinates correspond to the sequences of the individual contigs, rather than the scaffold as a whole. Figures in parentheses indicate the reading frame in which the ORF lies.

^3^ This column shows the best matches obtained by BLASTP searches of the protein sequences in GenBank using each ORF sequence as a query. Listed are the protein identification number of each best match, a description of the protein, its host, and the E-value of the match. 'No match' indicates either that no matching sequence was identified or that the E-value of the best match was greater than 1.

^4^ This column shows the best matches obtained by TBLASTN searches of the *P. sojae* genome sequence database using each ORF sequence as a query. The scaffold number, the coordinates of the match and the E-value are listed. 'No match' indicates that no matching sequence was found.
